# The National Medical Guild: The First Success of the New Trade Union

**Published:** 1913-05-24

**Authors:** 


### THE NATIONAL MEDICAL GUILD.

### The First Success of the New Trade Union.

CONSIDERABLE interest in this Guild has arisen amongst members of the profession, and some impatience has been expressed that full particulars of it, with application forms to join, have not been sent to every member of the profession. We understand the delay to be due to a technical difficulty owing to the time it has taken to complete the registration of this Guild under the Trades Union Act. The present position is set forth in the following communication, dated 15th instant, signed by Mr. Gordon R. Ward, the organising secretary, which we have just received:—

SIR,—At a meeting of the Council of the National Medical Guild held on May 7 it was decided to ask you to publish the following particulars of the Guild:—

The Guild is a trade union for medical practitioners, and is run on trade union lines—*i.e.*, its main policy is the defence of its members by all legal means.

It has, of course, as a trade union, immunity from many actions under the conspiracy and libel laws; this is absolutely essential for the protection of its funds and for the success of any combined action on the part of the medical profession, and such immunity can only be obtained by a trade union.

It has already successfully contested the case of Heard *v.* Pickthorne, with the result that it is now illegal for societies to refuse non-panel doctors' certificates for the purposes of sickness benefit. This was a very extensive and injurious practice previous to this decision.

It is establishing branches in all parts of the United Kingdom.

The subscription is £1 1s. per annum, and cheques should be made payable to the National Medical Guild. Medical practitioners are eligible whether on the panel or not, and, in view of the fact that the Government are even now considering the inclusion of dependants of the insured under the Act in the provisions for medical benefit, it is hoped that those who have not previously had an opportunity of joining will do so now. The terms which the Government have under consideration are such that it is certain that every effort of the profession will be required to ensure any approach to reasonable terms.—I am, Sir, yours faithfully,

GORDON R. WARD,

Organising Secretary.

Temporary Offices:
34 Villiers Street, Strand, W.C., May 15, 1913.

